# Mathematical achievement: the role of spatial and motor skills in 6–8 year-old children

**DOI:** 10.7717/peerj.10095

**Published:** 2020-10-06

**Authors:** Laura M. Fernández-Méndez, María José Contreras, Irene Cristina Mammarella, Tommaso Feraco, Chiara Meneghetti

**Affiliations:** 1 Department of Psychology, Universidad Rey Juan Carlos, Madrid, Spain; 2 Department of Basic Psychology I, Universidad Nacional de Educación a Distancia, Madrid, Spain; 3 Department of Developmental Psychology and Socialisation, University of Padova, Padova, Italy; 4 Department of General Psychology, University of Padova, Padova, Italy

**Keywords:** Mathematical achievement, Mental rotation, Spatial cognition, Motor ability, Primary school children

## Abstract

Several studies have tried to establish the factors that underlie mathematical ability across development. Among them, spatial and motor abilities might play a relevant role, but no studies jointly contemplate both types of abilities to account for mathematical performance. The present study was designed to observe the roles of spatial and motor skills in mathematical performance. A total of 305 children aged between 6 and 8 years took part in this study. A generalized linear regression model with mathematical performance as a dependent variable was performed. Results revealed that Block design (as a visuospatial reasoning measure) accounted for mathematical performance, especially among 6- and 7-year-olds but not in 8-year-olds. After controlling for the effect of the block design, mental rotation and manual dexterity predicted mathematical performance. These findings highlight the role of underlying cognitive (spatial) and motor abilities in supporting mathematical achievement in primary school children.

## Introduction

Mathematical skills are related with socioeconomic status across lifespan, given that poor mathematical competence is linked with lower qualifications in academic curricula and low socioeconomic attainment (Duncan et al., 2007; Ritchie & Bates, 2013). In fact, it is estimated that approximately 17% of compulsory instruction is dedicated to mathematics in primary school (OECD, 2017), reflecting the critical importance of math skills in early developmental stages. Therefore, mathematical outcome has been extensively studied to investigate what factors underpin its attainment in primary school children, where math starts to be acquired and will be implemented in successive years, as it is a predictor of academic achievement (Duncan et al., 2007). Several factors can concur in mathematical skills acquisition and among them spatial and motor abilities seem to play a key role in this domain (e.g., Fernandes et al., 2016; Mix et al., 2016; Tam, Wong & Chan, 2019). However, to our knowledge, there are no studies that contemplate both domains to account for mathematical performance. Thus, the main aim of the current study is to examine the contribution of both spatial and motor abilities to mathematical performance in primary school children.

### Relations between spatial skills and mathematical performance

Spatial ability, defined as “the capacity to create, retain, retrieve and transform well-structured visual images” (Lohman, 1996, p. 112) is the basis of spatial thinking (Hegarty & Waller, 2005), which will subsequently help in performing tasks in domains that are not, on the surface, obviously spatial. With respect to academic success, a variety of studies have found a positive relationship between spatial abilities and mathematical performance from early childhood to adulthood (Delgado & Prieto, 2004; Gunderson et al., 2012; Höffler, 2010; Lubinski, 2010; Mix et al., 2016; Thompson et al., 2013; Verdine et al., 2017), also in atypical populations (e.g., Mammarella et al., 2013a; Mammarella et al., 2013b; Passolunghi & Mammarella, 2012).

Some authors have suggested that an explanation for this relationship is that numerical mental models may be grounded on spatial representations (Hawes, Tepylo & Moss, 2015a; Verdine et al., 2014). Among the variety of spatial skills (Uttal et al., 2013), a particular role seems to be played by Mental Rotation (MR). Uttal et al. (2013) proposed a model of spatial skills, based on the intersection of two fundamental dimensions. The first dimension refers to intrinsic vs. extrinsic abilities, being the intrinsic information related with the specification of the object parts and the relation among them, and the extrinsic information referring to the relation between objects and context. The second dimension involves dynamic vs. static abilities, with dynamic abilities being based on the transformation of a stimulus and static abilities being based on retaining a static presentation. These dimensions are arranged in a 2 ×  2 model with four possible combinations (intrinsic-static, intrinsic-dynamic, extrinsic-static and extrinsic-dynamic). In this sense, MR would be categorized as an intrinsic-dynamic ability (Uttal et al., 2013), as it requires rotating (dynamic) two or three-dimensional objects (intrinsic) in the mind (Linn & Petersen, 1985).

MR ability has been proposed as a ‘cognitive tool’ involved in mathematical representation, where numbers and other elements should be mentally represented and rotated to accomplish various types of mathematical requests. Specifically, MR is related to arithmetic and mental calculation (Cheng & Mix, 2014; Kyttälä & Lehto, 2008; Thompson et al., 2013). MR is also related to rotating geometric pieces and learning about polymers (Wu & Shah, 2004), visualizing length or sizes (Delgado & Prieto, 2004), or composing and decomposing 2D and 3D figures, proving symmetry (Cheng & Mix, 2014).

The relationship between MR and mathematical ability could vary according to age and the type of mathematical task. Longitudinal developmental studies have shown that spatial performance (involving MR and Block Design, an intelligence measure based on visuospatial reasoning that involves analyzing, synthesizing and manipulating spatially abstract visual stimuli) in 1st grade children predicted success in different types of mathematical problems 4 years later, specifically in spatially-related math reasoning problems, verbal analytical reasoning, and arithmetic (even if the sample was only composed by females; Casey et al., 2015; Casey et al., 2017).

In a cross-sectional study involving more than 800 children aged between 5 and 13 years, Mix et al. (2016) tried to clear up the spatial-mathematical relationship. Spatial abilities were measured through different constructs, such as MR, visuospatial working memory, visual motor integration, Block Design, map reading and perspective taking. These authors measured MR through a rotation task that requested identifying the two rotated correct alternatives that represent the target item using 2D forms for kindergarten and third grade children, and 3D block construction for the sixth-grade children (based on an adaptation of Neuburger et al. (2011), and Peters, Chisholm & Laeng (1995) tasks). Visuospatial working memory was assessed through a task where the child had to indicate the sequential correct positions of one object that he/she had observed previously on a grid. Visual motor integration, or the ability to perceive and accurately copy simple forms by drawing them was assessed following Beery & Beery’s design (2010). Block Design was used to measure the ability to solve part-whole relation tasks, while the child rotated small blocks to reproduce the correct pattern (Wechsler et al., 2004). Map reading, which involves scaling ability, was assessed by measuring the ability to map differently sized representations onto each other by mentally transforming their extent, following Liben & Downs (1989) design. Perspective taking, or the ability to visualize objects from the vantage point of an imaginary observer at some other point in space, was measured according to Frick, Möhring & Newcombe’s (2014). According to developmental ages, Mix et al. (2016) showed that spatial and mathematical skills were different constructs that were highly correlated. In particular, in kindergarten, MR and Block Design were strongly related to mathematical ability. In sixth grade, a different pattern emerged. Visuospatial working memory (i.e., the ability to maintain and process visuospatial information; Logie, 1995), visual motor integration and perspective taking were strongly related to mathematical ability, such as algebra and place value, but not Block Design or MR. It is interesting to note that these authors differentiated their results according to new content - items related to content from the children’s current grade level or higher- and familiar content - items related to content from any of the preceding grade levels- in relation to age according the standards of the CCSS-M (National Governors Association Center for Best Practices and Council of Chief State School Officers, 2010) and found a different involvement of abilities in relation to age. Specifically, when the material was familiar, MR was a predictor in kindergarten and third grade, but not in older children, whereas visuomotor integration was a predictor in all three grades. Also, visuospatial working memory and perspective taking were predictors only in third and sixth grades, although Block Design did not predict math performance with familiar content in any of the courses. When the material was new, MR showed the same results, as a predictor in the younger children (kindergarten and third grade), but not in older children. However, Block Design showed a reversal pattern with respect to familiar content, being a predictor in all three grades. Moreover, visuospatial working memory was a predictor in third and sixth grades, and visuomotor integration for older children, whereas perspective taking was a predictor for kindergarten children. According to the authors, children seem to recruit spatial visualization and rotation when the content of math tasks are new and when they require grounding or conceptualization. Contrastingly, when the task becomes more automatic, such as reading symbols in sixth grade, performances depend on form perception. It seems that all abilities, spreading across visuospatial aspects (working memory, visuo-construction, MR), are involved in both types of material but their involvement is age-related, with specific spatial visualization involvement when the content is familiar.

In another study, Delgado & Prieto (2004) demonstrated that MR predicted geometry and word problems (which involve a written text containing a set of data followed by one or more questions) but not arithmetic performance in 13-year-old children. Recently, Hawes et al. (2019) found fresh evidence that spatial abilities (visual-spatial reasoning, 2D mental rotation and raven’s matrices) and numerical abilities (including ordering, symbolic and non-symbolic comparison tasks) predicted math achievement using tasks based on counting, ordering, operations, place value, fractions/proportions/decimals, in primary school children aged between 4 and 11 years.

Other research has considered mediating variables in studying the relationship between spatial abilities and mathematical achievement. Gunderson et al. (2012) found that MR at the beginning of 1st and 2nd grades predicted improvement in number line representation over the course of the school year. Furthermore, the relationship between young children’s early spatial skills and their later mathematical achievement (i.e., age 8) was mediated by the children’s early skill at number line representations. In a further study with 2nd grade students, Tam, Wong & Chan (2019) demonstrated that mental number representation fully mediated the relationship between rotation skills (in terms of rotating objects–MR–and assuming image rotation views–perspective taking) and calculation as well as word-problem solving. This type of results indicates that it may be relevant to take into account the type of rotation requests in relation to math performance.

Regarding this concern, in a longitudinal study from kindergarten to second graders, Frick (2018) showed through structural equation models that different rotation abilities, in terms of assuming image rotation (such as perspective taking) and imagery rotating objects (such as MR) differently predict performance in the type of math tasks. In particular, the perspective taking ability showed the strongest relation to the part of the math test that tapped into arithmetic operations, whereas MR was strongly related to Numeric-Logical and Spatial Functions, as well as geometry. These findings revealed that rotation abilities with different proprieties are involved in mathematical performance and their role can change as a function of the type of task in relation to age.

Overall, this brief review of the literature supports evidence of the relation between spatial rotation ability and several types of mathematical tasks from an early age (Casey et al., 2015; Casey et al., 2017; Mix et al., 2016). This relationship changes as a function of age, type of math tasks and also, type of rotation measures used (subjective vs. objective-centered; (Frick, 2018). However, previous research has not tested the relation between spatial (including MR) abilities and math over and above the role of visuospatial reasoning. In some of the previous studies, Block Design, a measure of visuospatial reasoning strongly related to math achievement (Kyttälä & Lehto, 2008; Passolunghi, Vercelloni & Schadee, 2007) has been used as a measure of spatial skills (Casey et al., 2015; Casey et al., 2017; Mix et al., 2016). Block design is a task typically used to measure visuospatial reasoning (Carroll, 1993) which involves both visualization (to imagine how blocks can be arranged) and constructive skills (to physically detect how to arrange blocks). This ability is not rotation-based, although it is related to rotation. Moreover, some authors have suggested that fluid intelligence is a separate construct to—although highly correlated with—spatial intelligence (composed by rotation measures; Martínez et al., 2011) with evidence that MR is the best predictor of fluid intelligence, explaining 23% of the variance of the fluid intelligence score among 15 and 16 year-olds (Kyttälä & Lehto, 2008). Therefore, from this evidence, it is important to examine the contribution of intervening variables to explain the role of visuospatial reasoning.

### Relations between gross- and fine-motor skills and mathematical performance

Several studies have shown a relationship between motor proficiency and an advantage of academic achievement across a variety of subjects (e.g., math, reading, written and oral language, biology, geography, physics, chemistry, visual arts, physical education, arithmetic, geometry, probability problems, among others) between the ages of 5 and 18 years (Carlson, Rowe & Curby, 2013; Davies et al., 2016; Diamond, 2010; Geertsen et al., 2016; Haapala et al., 2014; Kantomaa et al., 2013). In fact, the evidence shows that the amount of learning that one child can acquire can be constrained by the child’s own motor abilities (Dejonckheere et al., 2014).

In this regard, Bornstein, Hahn & Suwalsky (2013), in a 14-year longitudinal study, showed that infants who had better motor control ability and who explored their environment more actively at 5 months obtained higher cognitive scores (assessed with intelligence measures) at the age of 4 and 10 years and also had a better performance in academic achievement tests in a variety of subjects at the ages of 10 and 14 years. The authors suggested that motor-exploratory competence in infancy initiates a developmental cascade that could affect subsequent levels of child intellectual functioning that, in turn, help shape academic achievement in adolescence. The results of this study indicated that motor-exploratory competence can establish the basis for subsequent cognitive functioning in childhood and academic achievement in adolescence.

Therefore, the role of high motor skills (e.g., manual dexterity or balance) in relation to high-quality performance of cognitive tasks also seems clear in related school subjects. The focus of the current study is, however, to examine whether the motor skills role can be detected also (or specifically) for mathematical ability. Mathematical tasks, especially in the first years of primary school, are based on concrete and practical tasks requiring to manually move objects and body movements to understand mathematical principles, for example, arranging cardboards depicting numbers in ascending order, placing in boxes common objects and discarding the non-common ones.

Motor skills are defined as learned sequences of movements that are combined to produce a smooth, efficient action in order to master a particular task (Davis, Pitchford & Limback, 2011). Motor skills are classically distinguished between gross motor skills that involve the large, force-producing muscles of the trunk, arms, and legs (Clark, 1994), such as throwing, catching or jumping (Logan et al., 2012), and fine motor skills, defined as movements that require mostly smaller muscles or muscle groups of the body (Payne & Isaacs, 2008), such as manual dexterity (Logan et al., 2012). Also, motor proficiency, as described by Bruininks (2005), incorporates the following components: fine motor precision, fine motor integration (visual motor integration), manual dexterity, as a component of fine motor skills; and upper limb coordination, bilateral coordination, balance, speed and agility, and strength as a component of gross motor skills.

In particular, fine motor skills have been reported as predictors of mathematical achievement in tasks such as those related with number and shape, relative size, ordinality and sequence, addition and subtraction, and multiplication and division (Grissmer et al., 2010; Luo et al., 2007). In a longitudinal study, Kim et al. (2018) found that fine motor coordination at the beginning of kindergarten is linked with mathematics skills (such as numeration, measurement or geometry measures) at the end of first grade (although this relationship is mediated by visuo-motor integration), showing how fine motor proficiency contributes to academic achievement as soon as children begin formal reading and math learning.

Moreover, gross motor skills, such as motor coordination of the body, have a role in school achievement, including math. Children of 9–12 years of age with low scores in motor coordination (such as balance, jumping laterally, or shifting platform) have a lower probability of obtaining general academic achievement with respect to children with normal motor coordination scores in 4th grade students (Lopes et al., 2013). Another study showed that children with learning disabilities showed worse performance in gross motor skills and academic success than their 7- to 12-year-old peers without learning disabilities (Westendorp et al., 2011).

Gross motor skills, such as motor coordination, are also directly related to arithmetic skills, in tasks where the examiner verbally presents a math operation and the child must write down the answer, among 8- to 14-year-old children (Fernandes et al., 2016). Whereas balance skills, in 6-year-old children, are related to spatial and reasoning skills in mathematics one year later (Frick & Möhring, 2016).

Other studies have considered at the same time both types of fine and gross motor skills and both have been related—although moderately—with performance in mathematics (arithmetic such as addition, subtraction and multiplication, geometry and probability problems) in children aged between 8 and 10 years (Geertsen et al., 2016), and even in preschoolers (Cameron et al., 2012).

Moreover, and as for spatial ability, the role of different motor skills can change as a function of age. In fact, Morales et al. (2011) showed how different motor skills were related with calculation ability regarding age range. In their study, mathematical ability (measured in terms of calculations, addition, subtraction, multiplication, and division, simple problems, and geometrical problems) was predicted by fine and gross motor skills in children aged between 9 and 12 years, whereas for children aged between 13 and 16 years, fine motor ability remained as a strong predictor and gross motor ability was no longer a significant predictor. In this vein, Da Silva Pacheco et al. (2016) highlighted the need to disentangle whether some motor skills have a stronger influence than others on academic achievement, showing initial evidence of their differential role on academic achievement in children (Da Silva Pacheco et al., 2016; Fernandes et al., 2016).

Overall, there is evidence that motor skills play a role in a variety of school subjects at early ages (e.g., Bornstein, Hahn & Suwalsky, 2013), including math. In fact, when fine (Kim et al., 2018) and gross (Fernandes et al., 2016) motors skills are examined together, their impact on mathematical achievement can change as function of age, with a prevalence of fine motor skills (Morales et al., 2011). According to the current state of affairs of the existing literature, it is not possible to make inferences regarding the different roles played by fine and gross motors skills in math in primary school. At the same time, given the recognized relation between motor skills and general functioning (such as intelligence measures; (Bornstein, Hahn & Suwalsky, 2013), it is not possible to disambiguate how motors skills cooperate with other cognitive abilities.

### Relations between motor skills and spatial abilities

Motor skills have been associated with spatial ability. Several studies have supported the role of motor skills in spatial reasoning tasks (Amorim, Isableu & Jarraya, 2006; Steggemann, Engbert & Weigelt, 2011; Wraga et al., 2003). Specifically, several studies have showed the relation between motor ability and MR in adults (e.g., Moreau, 2012; Wexler, Kosslyn & Berthoz, 1998) and children (Frick et al., 2009; Krüger & Krist, 2009). Motor action has a supporting effect in MR (Chu & Kita, 2011; Ehrlich, Levine & Goldin-Meadow, 2006). Funk, Brugger & Wilkening (2005) showed that MR is predicted by body movement through the mediation of motor skills. The impact of motor abilities on spatial ones is also demonstrated by the positive effect of motor training on MR performance (Wiedenbauer & Jansen-Osmann, 2008). Regarding children, Jansen, Lange & Heil (2011) demonstrated an improvement in a MR task in girls aged 6 to 14 years old after three months of motor ability juggling training in comparison to those children that trained with thera-band stretch bands. In the same vein, Blüchel et al. (2013) trained 8 to 10 year old children in motor coordination (high ratio of velocity, endurance and coordinating challenge) daily during two weeks and showed a significantly higher increase in MR abilities compared to the control group without physical activity.

These findings showed that the relationship between MR and motor ability is well established across development, from preschoolers (Jansen & Heil, 2010; Lehmann, Quaiser-Pohl & Jansen, 2014), to primary school children (Jansen, Lange & Heil, 2011) and even to adults (Voyer & Jansen, 2017), reflecting the strong association between them.

### Rationale of the current study

The current study was based on these premises: (i) previous studies have shown relations between spatial skills and performance in mathematics, where MR can assume a particular role even at early ages (e.g., Mix et al., 2016; Thompson et al., 2013; Zhang et al., 2014). Given that this relation can be mediated by several variables, it is not fully clear whether MR has a distinctive role on math performance after controlling for the general cognitive functioning, expressed in terms of visuospatial reasoning. (ii) Previous research has revealed a relation between motor abilities and academic school achievement (Bornstein, Hahn & Suwalsky, 2013) with a detectable role of both fine (Kim et al., 2018) and gross (Fernandes et al., 2016) motors skills. It appears unclear, however, whether fine and gross motor skills have a different role in primary school mathematical achievement and, given its relationship with visuo-spatial reasoning (Bornstein, Hahn & Suwalsky, 2013), to what extent it cooperates with other cognitive abilities.

Based on these premises, the present study aims to jointly examine the contribution of both spatial (in term of MR) as well as different motor (fine and gross) skills to mathematical performance (specifically, written arithmetic calculation and number ordering tasks) in primary school children. We assessed mathematics abilities in a large sample of primary school children between grades 1 and 3 (6-, 7-, and 8-year-olds). As for the mathematics tasks, we used written calculations and number ordering. Arithmetic or calculation-based tasks have been frequently used by most studies as an index of mathematic performance (e.g., Filippetti & Richaud , 2017; Hawes et al., 2019; Kyttälä & Lehto, 2008; Mix et al., 2016), being these type of tasks essential in the core of mathematics education for the elementary grades according to the National Council of Teachers of Mathematics (NCTM, 2000). In the number ordering task, the numbers are processed as ordered sequences, one important marker of an individual’s basic number skills (Hawes et al., 2018), where a positive relation between children and adults’ ordinality skills and mathematical performance has been found (Lyons et al., 2014; Lyons, Vogel & Ansari, 2016). In fact, Ontario Mathematics curriculum (grades 1-8) includes arithmetic and ordering number tasks in Number Sense and Numeration strands (Ontario, 2005). Furthermore, in our study, children also performed MR and motor tasks. For motor skills, we assessed Manual Dexterity (as fine motor skills), Throwing and Hold and Balance skills (as gross motor skills). As a control, a measure of visuospatial reasoning, the Block design task, was also administered.

Given the previous evidence of the contribution of spatial abilities (as MR) in math performance (e.g., Mix et al., 2016; Tam, Wong & Chan, 2019), we expect that MR can be a relevant predictor of mathematical performance in primary school children. Furthermore, given that MR and visuo-spatial reasoning are related (Bornstein, Hahn & Suwalsky, 2013; Casey et al., 2015; Casey et al., 2017), we decided to control for visuo-spatial reasoning skills, to test the unique role of rotation ability as a predictor of mathematical performance.

In addition, we examined whether the roles of fine and gross motor skills are similar or whether one prevails over the other (as suggested by Morales et al., 2011; a prevalence of fine motor skills with increasing age). Moreover, given that motor skills are related to visuo-spatial reasoning (Bornstein, Hahn & Suwalsky, 2013) and also to spatial ability, in young adults (Voyer & Jansen, 2017) primary school children (Frick & Möhring, 2016; Pietsch, Böttcher & Jansen, 2017) and preschoolers (Lehmann, Quaiser-Pohl & Jansen, 2014), we examined whether it is possible to detect the specific role of motors skills after controlling for the cognitive abilities (as visuo-spatial reasoning and rotation ones).

## Material and Methods

### Participants

A sample of 305 primary students (162 males and 146 females) of seven different public schools (located in urban areas with similar characteristics) from northern Italy took part in this study (78 six year-old participants; 35 F, mean age = 6.56, SD = 0.27; 112 seven year-old participants; 48 F; mean age = 7.50, SD = 0.28; and 115 eight year-old participants; 61 F; mean age = 8.55, SD = 0.34). Children were recruited from local schools through a letter explaining the study (aim, method and requests). After accepting to participate in the research, the schools took care of asking for the consent of the students’ parents. Written informed consents from participants were collected. The study was approved by the Research Ethics Committee of University of Padova (number: 8B7B34CA379BBD8A0AA069C2E4B0F0FC).

### Materials

*Mental rotation task* (adapted from Jansen & Kellner (2015)). This task measures the ability to rotate mental representations of two-dimensional and three-dimensional objects in the mind. The stimuli consisted of seven different animal pictures (duck, camel, mouse, donkey, sheep, dog, and rabbit). Three stimulus pairs were used for the practice trials to familiarize children with the task, and four different stimulus pairs were used for the experimental trials. The test was composed by 5 practice trials (angular disparities: 0°, +45°, −90°, +135°, 180° ), and 32 experimental trials with the following angular disparities: 45°, +90°, +135°, 180°, −135°, −90°, and −45°) with 4 items for each angle, except 180°, which was composed of 8 items. The setup was the same for all children, regardless of their dominant hand. This test showed a good internal consistency (current sample *α* = 0.76).

The test was set up using E-Prime (version 2.0) software and projected on a laptop computer (17 inch). Two stimuli were presented on the screen simultaneously. The left stimulus always appeared in an upright position. The right stimulus was identical to the left stimulus or mirror-reversed. A positive angle corresponded to the stimulus being rotated in a clockwise direction and a negative angle corresponded to the stimulus being rotated in a counterclockwise direction. The task consisted in deciding whether the two stimuli on the screen were the same or mirror reversed by pressing one of two marked keys on the keyboard of the laptop. Participants gave their answers by pressing the “M” or “Z” keys on the keyboard for “Same” or “Different”, respectively. In the instructions, it was specified to mentally rotate the right stimulus to align it with the left, upright stimulus. One point was awarded for each correct response, with a maximum total score of 32 points.

*Block Design* (from Wechsler Intelligence Scale for Children, WISC-IV; Wechsler et al., 2004; Italian version by Orsini, Pezzuti & Picone, 2012). This test measures the ability to analyze and synthesize abstract visual stimuli by spatially manipulating them. It can be applied to children aged between 6 years and 16 years and 11 months. The material consists of 9 blocks (with two red, two white and two bicolor red-white sides) and one notebook which has images constructed with the blocks. There are 14 items of increasing difficulty, i.e., using 4 (from item 4 to 10) or 9 (from item 4 to 14) blocks within a given time limit. The time limit varied in relation to the items, with 30 (item 1), 45 (items 2 to 5), 75 (items 6 to 10), or 120 s (items 11 to 14) to build the figure. For the first three items, the participants had to reproduce a model made by the experimenter, with two attempts for each item. For the following items, the participant had to reproduce the image shown in the notebook. For the three first items, one or two points were awarded according to whether the participant reproduced the model in the first or second attempt, respectively, in 30 s for first and second items, and 45 s for third item. For items 4 to 8, 4 points were awarded when the participant reproduced the image in the established time (45 s for items 4 and 5, and 75 s for items from 6 to 8). For items 9 to 14, 4, 5, 6 or 7 points were awarded for each item depending on the time spent solving each item. The maximum score was 68 points (see Table S1). The task ended when the child failed and obtained two consecutive scores of 0 points. The raw scores were converted into standard scores following the Italian norms (Orsini, Pezzuti & Picone, 2012). This test showed a good internal consistency (*α* = .82 WISC-IV; Italian adaptation, Orsini, Pezzuti & Picone, 2012).

*Movement Assessment Battery for Children –Second Edition* (MABC-2; Henderson, Sugden & Barnett, 2007; Italian version by Biancotto et al., 2013). This battery aims to identify the movement ability of children and adolescents aged between 3 and 16 years to evaluate their motor coordination in terms of Manual Dexterity, Aiming and Catching, and Balance. Based on the suggestions of the manual, different tasks were used according to the age of participants (age range 1: children from 4 to 6 years old; age range 2: children from 7 to 10 years old).

Manual Dexterity measures fine motor skills and is composed of three tasks: Insert Coins, Thread Beads and Drawing a Trail 1 for age range 1; Placing Pegs, Threading Lace, and Drawing a Trail 2 for age range 2. For age range 1, the insert coins task consists in inserting coins as fast as possible through a small slot into a box while holding this box with the other hand. In the Thread Beads task, the participant is advised to thread beads as quickly as possible. When performing the task, one hand is holding the string, while the other hand is threading the beads. The Drawing Trail 1 task consists of drawing a line following a pathway. In the age range 2, the Placing Pegs task consists in inserting pegs into holes within a pegboard. The Threading Lace task consists of inserting a cord through six holes aligned on a lacing board. The Drawing Trail 2 task consists of drawing a line following a pathway.

The Aiming and Catching test measures gross motor skills and is composed of two tasks: Throwing a Beanbag and Catching a Beanbag for age range 1; Catching with Two Hands and Throwing Beanbag onto Mat for age range 2. In the age range 1, in the Throwing a Beanbag task, the participant is standing on one of the mats and must throw the beanbag onto a circle that is positioned in front of the participants. In the Catching a Beanbag task, the participant is advised to catch the sandbag. In the age range 2, Catching with Two Hands consists of throwing a tennis ball towards a wall from a distance of 2 m and catching it with two hands. The Throwing Beanbag onto Mat task requires throwing a little bag onto a mat with a target placed at 1.8 m of distance.

The Balance skills test measures gross motor skills and is composed of three tasks: One Leg Stand, Walking with Heels Up, and Mat Bouncing for age range 1; One-Board Balance, Walking Heel to Toe Forwards, and Hopping on Mats for age range 2. In age range 1 the one Leg Stand task consists in standing on one leg for as long as possible. In the Walking with Heels Up task, the participants were asked to walk on a fixed line with heels up as exactly as possible. In the Mat Bouncing task, five mats are positioned against each other and on each mat the participant needs to jump once with both feeds. In age range 2, in the One-Board Balance task, participants were asked to maintain their balance on one leg on top of a board (there are two attempts for each leg). The Walking Heel to Toe Forwards task involves walking over a strip of adhesive tape 4.5 m long, placing the heel of one foot on the tip of the other at each step (there are two attempts). The Hopping on Mats 2 task consists in jumping forward with one leg onto six mats placed in a row until they reach the target mat, characterized by an orange circle (there are two attempts for each leg).

For each test item, the experimenter recorded different measures regarding the test, i.e., time in seconds for Insert Coins, Thread Beads, Placing Pegs, Threading Lace; Numbers of error for Drawing a Trail 1 and Drawing a Trail 2; number of correct catches/throws out of 10 for Throwing a Beanbag, Catching a Beanbag, Catching with Two Hands and Throwing Beanbag onto Mat tests; number of seconds balanced for One Leg Stand and One-Board Balance tasks; number of correct steps for Walking with Heels Up and Walking Heel to Toe Forwards; and number of correct jumps/hops out of 5 for Mat Bouncing and Hopping on Mats 2. The total raw scores for each task were then converted into standard scores based on the normative data for each test (Italian norms; Biancotto et al., 2013). The Cronbach’s alpha coefficients for all domains ranged between .77 and .80).

*Mathematical achievement* (Battery AC-MT 6-11; Cornoldi, Lucangeli & Bellina, 2012). The battery measures mathematical skills in children aged between 6 and 11 years. From this battery, we selected the written arithmetic calculation and the number ordering subtests. The written arithmetic calculation subtest is composed of 4 items for first and second grade (2 additions and 2 subtractions) and 8 items for third grade (2 additions, 2 subtractions, 2 multiplications, and 2 divisions). Children have to solve each operation by using the correct procedure. The number ordering subtest requires placing in order 4 (for first grade) or 5 (for second and third grade) series of 4 numbers from the lowest to the highest and then, 4 digits ordered from the highest to the lowest. Children must resolve both tasks on an answer sheet provided by the experimenter. One point was awarded for each correctly solved operation or each correctly completed series. The raw scores were converted into standard scores following the Italian norms (Cornoldi, Lucangeli & Bellina, 2012). The test-retest reliability scores were .74 for the written calculation and .68 for the number ordering.

### Procedure

The tasks were administered in three sessions: two individual sessions and one collective session (30 min each one, approximately). While the first two sessions were carried out individually in a quiet room, prepared to prevent distractions and increase concentration, the collective session was performed in a classroom in presence of teachers.

In the first individual session, children completed, in this order, the mental rotations task and the MABC-2 tests. In the second individual session, they were presented with the *Block Design* test*.* In the third session, children were presented with the AC-MT 6-11 battery.

## Results

### Preliminary analyses

Descriptive statistics and correlations between all variables are reported in Table 1.

**Table 1:** Descriptive statistics and Correlation Matrix between all the variables included in the research. Mental rotation mean and SD are in raw scores.

	M (SD)	1.	2.	3.	4.	5.	6.
1. Math	0.86 (0.18)	–					
2. Age	7.67 (.85)	.03	–				
3. Block Design Task	10.31 (3.54)	.25^*^	−.02	–			
4. Mental Rotation Task	23.57 (5.19)	.31^*^	.07	.21^*^	–		
5. Manual Dexterity Tasks	9.36 (2.69)	.30^*^	.21^*^	.34^*^	.14	–	
6. Aiming & Catching Tasks	7.52 (2.69)	.05	.14	.22^*^	.09	.19^*^	–
7. Balance Tasks	10.19(3.95)	.13	.33^*^	.07	.09	.17	.07

**Notes.**

* *p* ≤ .001.

It should be noted that in the sample two students were less of 6 year olds (i.e., 5.94 and 5.92). However, they were included in the analyses given that their performance resulted similar to 6 years old students’ performance.

Generalized linear regression models were carried out using the lme4 package (Bates et al., 2015) in R (R Core Team, 2018) to examine the predictors of mathematical scores. Both written calculation and number ordering were treated as a single score in the analyses because both indicate basic mathematical abilities needed for this school range and showed to be correlated between each other’s (*r* = .30 *p* < .01)^1^ 1The same analyses were performed considering the arithmetic calculation and the number ordering subtests separately and the results substantially overlapped.. Given that the scoring of Mathematical tasks was calculated by assigning one point for each correct answer and zero points for each wrong answer in both sub-tests (written calculation and number ordering), generalized linear regression models with mathematical performance as a binomial dependent variable were run.

In the models, Age was treated as a continuous predictor variable. For all the other predictors, the standardized scores according to the normative sample were used in the model except for the MR Task, for which the sample’s scores were used (given no normative sample was available).

The model selection was conducted on the basis of AIC (Akaike Information Criterion, Wagenmakers & Farrell, 2004), which gives a measure of out of sample deviance and enables comparisons between alternative models. Lower out of sample deviance produces lower AIC, which indicates that the model is better.

For each model, we also calculated the effect of the predictors with the likelihood-ratio tests based on chi-square distribution to confirm the AIC indication. The effect of each parameter was calculated through odds ratios, which correspond to the change in the odds given an increase of 1 point in the specific predictor variable (Bland & Altman, 2000).

### Predictors of mathematical achievement

The predictors were entered into the models starting from a baseline null (m0) including only the intercept (without any predictor).

In the first model (m1), Age was entered to examine its general effect on mathematical scores. In the successive models, we inserted, in this order, Block Design (m2), Mental Rotation (m3) and Motor skills (m4) tasks, each one, as a simple additive factor and taking into consideration their interaction with age to understand whether the effect of the factors remained constant or changed as a function of age.

Block Design was inserted in m2, as a visuo-spatial reasoning (intelligence) measure, to assess the effect of general functioning ability (given its relationship with mathematical performance, Deary et al., 2007; Moenikia & Zahed-Babelan, 2010). Then, m3 and m4 assessed the role of MR and motor skills (after controlling for the visuo-spatial reasoning) respectively. Inserting the variables in this order allowed to detect the specific role of MR without the effect of visuo-spatial reasoning, as the two abilities proved to be related and, when considered together, both emerged (Casey et al., 2015; Casey et al., 2017) or prevailed over the spatial -rotation- ones (Mix et al., 2016). This order of insertion also allowed to detect the role of motor skills after controlling for cognitive abilities, as motor skills are related to cognitive abilities — such as intelligence — (Bornstein, Hahn & Suwalsky, 2013) and spatial abilities (Frick & Möhring, 2016).

Therefore, we started with the model selection (values are reported in Table 2). Age (m1) produced negligible changes in values (AIC = 1528.33) in comparison to m0 (AIC = 1526.54). Furthermore, in m2, the addition of Block Design showed a decrease of AIC both when considered alone (AIC = 1455.91) and in the interaction with Age (AIC = 1433.38), producing a significantly better model ( *p* < .001). Therefore, the model selected included the Block Design score and its interaction with age and became the baseline (m2) for the successive models.

**Table 2:** Model selection process. The significant factors are highlighted in bold and were selected for the final model.

Model	Predictors	Resid. Df	Resid. Dev	Df	Deviance	*p*	AIC
m0		304	1,020.50				1,526.54
m1	+Age	303	1,020.25	1	0.243	.622	1,528.33
m2	**+Block Design**	**302**	**945.79**	**1**	**74.45**	**<.001**	1,455.91
	**+Age x Block Design**	**301**	**921.21**	**1**	**24.58**	**<.001**	1,433.38
m3	**+Mental Rotation**	**300**	**843.56**	**1**	**77.64**	**<.001**	1,357.80
	+Age x Mental Rotation	299	843.11	1	.457	.498	1359.42
m4	**+Manual Dexterity**	**299**	**789.18**	**1**	**54.38**	**<.001**	1,305.50
	+Age x Manual Dexterity	298	789.18	1	.001	.972	1,307.59
	+Aiming and Catching	298	786.71	1	2.47	.115	1,305.12
	+Balance	298	786.63	1	1.18	.278	1,305.04

In the third model (m3), the addition of MR as a single factor showed to be significant (*p* < .001) with decreasing AIC (AIC = 1357.80) but no significant contribution was detected when the interaction with age was included in the model (AIC = 1359.42; *p* = .49). Therefore, the selected model (m3) included only MR as a single additive factor.

In the successive model (m4), the three motor skills scores were considered: The Manual Dexterity score was added first, then Balance and finally, Aiming and Catching (according to the correlation values). If neither the additive nor the interaction model decreased the AIC value, compared to the specific baseline model, the predictor was not included, and the baseline model was maintained for successive comparisons. Therefore, in m4, only Manual Dexterity (AIC = 1305.50, *p* < .001) resulted a predictor of mathematical score as a single additive factor but no significant contribution was detected when the interaction with age was included in the model (AIC = 1307.59; *p* = .97). When Aiming and Catching and Balance scores were inserted into the model, slightly contradictory values of AIC and *χ*^2^ (*p* = .115, *p* = .109 respectively) were found, but given the small and non-informative difference between the AIC of the model with Manual Dexterity and the one with Aiming and Catching (ΔAIC = .38) or Balance (ΔAIC = .46), we decided not to include Aiming and Catching or Balance in the model.

The final model is therefore comprehensive of Age, Block Design, Mental Rotation, Manual Dexterity, and the interaction between Age and Block Design.

All of these parameters (see the values reported in Table 2) resulted as positive predictors (*p* <.001) of mathematical score except for the interaction between Age x Block Design, which showed to be significant but negative; this indicates a decrease of the effect of Block Design when age increases (OR = .932). It is noteworthy that the estimated effect of Age in the final model became significant likely because of the variance of age that was included in the significant interaction with Block Design.

## Discussion

The main goal of this study was to determine the contribution of MR and motor abilities (fine and gross skills) to mathematic achievement in children aged between 6 and 8 years. This developmental stage is critical in acquisition and consolidation of basic skills that help to build a foundation where mathematical ability can arise and even be promoted through the identification of the key factors that can underlie mathematical achievement. In fact, the competence shown in math at school entry is a predictor of academic success in later years (Duncan et al., 2007), highlighting the importance of determining which factors contribute to its achievement.

The assessment of the effect of general functioning ability in term of visuo-spatial reasoning –Block Design- across age, reveals its general role on mathematical performance and its different contributions as a function of age. The variance associated to mathematical success was greater in 6 year-old children, but it decreased its explanatory power in 7 year-olds, contributing very poorly in 8 year-old children. It seems that the relation between Block Design and mathematical performance varied as a function of age. In order to explain our result, it is plausible that at the beginning of school entry, visuospatial reasoning has a greater role in a variety of tasks, including mathematical performance yet, with increasing age, the development of other cognitive skills such as MR can have an increasing role in explaining math performance in later stages.

In fact, as expected, a specific contribution of MR on mathematic performance, after controlling for visuospatial reasoning, was observed. The MR ability –based on imagery rotating objects- requires detecting whether couples of objects are different (mirrored) or similar (assuming different orientation), and its contribution was clearly found after controlling for the visuospatial reasoning in all age groups. Indeed, in the regression models where Block Design was inserted before the MR task, the fact that MR emerged as a significant factor on mathematical performance, indicated that success in mathematical tasks is specifically explained by MR as a unique role, excluding the variance shared with visuospatial reasoning (Martínez et al., 2011).

The novelty of these results is, therefore, the emergent role of rotation ability, after controlling for general functioning, in mathematical achievement in primary school children. In this regard, the previous studies that examined the factors that underlie mathematical ability did not account for MR, after controlling for visuospatial reasoning skills - Block Design - (Delgado & Prieto, 2004; Frick, 2018; Gunderson et al., 2012; Hawes et al., 2019; Tam, Wong & Chan, 2019). This finding is particularly important given that it demonstrates that mentally rotating objects aids success in mathematical tasks, such as arithmetic calculation and number ordering at these early stages of primary education. These results are in line with other studies that have showed a correlation between MR and mathematical ability in 7 and 8 years old children (Heil & Jansen-Osmann, 2008). In this regard, (Kyttälä & Lehto, 2008) did not find that MR could explain the variance of arithmetic ability in children aged between 15–16 years old, possibly due to the common variance with measures of fluid intelligence (such as Raven Test or Visuo-spatial reasoning; Raven, Raven & Court, 1998). It seems that, at least, for children between first and third course of primary education, MR, excluding visuospatial reasoning, has a distinctive role in explaining mathematical performance. MR can support mathematical tasks based on ordering or missing term problems, as mental representations can be moved in the mind to change the position of the numbers to resolve both types of task successfully. This type of mental representation can have a distinctive role especially at early ages, which can be at the basis of other more complex mathematical competences (Gunderson et al., 2012). Therefore, when mathematical complexity increases, rotation and other abilities (including other fluid ones) can all aid in mathematical success. This needs to be better examined by enlarging the age range, type of mathematical tasks and cognitive abilities studied.

The possibility that MR is by itself an underlying factor to mathematical success allows proposing specific MR trainings to impact directly on mathematical achievement in the first courses of Primary Education. In fact, MR training has demonstrated its effectiveness at different stages of the educational cycle, such as among preschoolers (Fernández-Méndez, Contreras & Elosúa, 2018; Fernández-Méndez, Contreras & Elosúa, 2020), and in Primary (Rodán et al., 2016) and Secondary School children (Rodán et al., 2019). However, the question of whether transfer to other abilities not directly trained (outside of spatial abilities) occurs remains unclear. Although Uttal et al. (2013) showed transfer effects of spatial training, particularly, in MR, studies on this matter are scarce. There is only one study that established an improvement on missing term problems after a single session of MR training in 6 to 8 year-old children (Cheng & Mix, 2014), while others failed (Fernández-Méndez, Contreras & Elosúa, 2018; Hawes et al., 2015b; Rodán et al., 2016; Rodán et al., 2019). Taking into account these findings, it would be interesting for future research, to analyze the effect of different MR trainings on several mathematical tasks across different age groups, starting with earlier ages.

Concerning the contribution of motor skills, we found that Manual Dexterity, a fine motor skill that consists on the ability to make coordinated hand and finger movements to grasp and manipulate objects (Makofske, 2011), was a predictor of mathematical performance. However, Aiming and Catching and Balance factors did not add explicative power in the final model. In other words, in the final model, after controlling for visuospatial reasoning and rotation abilities, fine motor skills, in terms of Manual Dexterity, emerged as a distinctive factor in predicting mathematical performance. This result is partially in line with previous studies, where fine motor skills (such as Manual Dexterity) were related to mathematical skills, such as addition and subtraction, or ordinality and sequence (Grissmer et al., 2010; Luo et al., 2007) in children aged 5 and 6 years (Kim et al., 2018). Therefore, it is particularly relevant to find how fine motor ability emerges as a predictor after controlling visuospatial reasoning and MR, as fine motor ability generally includes strong spatial skills components (Verdine et al., 2017).

Contrary to other previous studies (Fernandes et al., 2016; Frick & Möhring, 2016), our results do not support gross motor skills, such as Aiming and Catching or Balance, explaining mathematical performance. However, Morales et al. (2011) showed that fine motor skills remained constant in predicting math among 9 to 16 year-old children, while gross motor skills decreased its prediction power. One possible explanation for these mixed findings can be that Manual Dexterity contributes strongly to mathematical tasks related with written calculations and number ordering, while gross motor skills are less related with these types of tasks. Differently, other mathematical tasks, such as reasoning skills may be more related with gross motor skills, such as Balance (Frick & Möhring, 2016). The result showing the role of fine, but not gross motor skills, in early ages in written calculation and number ordering is surely interesting but needs to be better consolidated in future studies examining the role of both fine and motor skills (given the evidence of their involvement; Fernandes et al., 2016; Frick & Möhring, 2016) in relation to other mathematical requests of increasing complexity (such as math problems).

The positive correlations found between MR and Manual Dexterity are noteworthy. This type of relation is consistent with previous evidence showing the link between the spatial rotation ability and motor skills (Voyer & Jansen, 2017; Frick & Möhring, 2016), where both share common neural resources, such as motor areas (Parsons et al., 1995). This result supports the embodied cognition approach (Wilson, 2002), linking motor processes and mental transformation, where sensory and motor functions would be involved in cognition. Specifically, the physical rotation of an object needs to involve fine motor movement, as it is an object manipulation action that does not involve egocentric movement, that would imply to move oneself (Voyer, Jansen & Kaltner, 2017), and would link it to a greater extent with gross motor abilities. In addition, it has been suggested that MR ability is an underlying process where individuals rotate the physical object into congruence in their minds (Wohlschläger & Wohlschläger, 1998). Probably, the MR of one representation is associated with the fine motor movements necessary to rotate this object in the real environment, connecting in this way MR and Manual Dexterity, aside from the motor areas implied in both abilities.

In relation to the limitations of the present study, it is necessary to consider that the results are relevant with the mathematical task used (written calculations and number ordering), and it may not be replicable with other mathematical problems that could involve other cognitive factors or not require MR ability or motor skills. For example, Hawes et al. (2017), in their intervention with children aged between 4 and 7 years old, only observed a transfer of a spatial ability training towards a basic numerical processing task (symbolic magnitude comparison) but not towards other calculation skills after 32 weeks of training. They separated the mathematical tasks they applied, into number-based and geometry questions. This analysis revealed that the improvement occurred only for the items linked to geometry-measurement concepts, but not to the number concept. So, the type of tasks and math content seems a sensible question for results obtained in studies that analyze relationships between spatial abilities and mathematical achievement.

Also, the order of execution of the different tasks administered may have influenced the results obtained. Specifically, a possible training due to execution in the MR task may have influenced the results of MABC-2 battery because MR was administered immediately before the MABC-2 battery. However, the other tasks were administered in different sessions and different days (block design in the second session and AC-MT 6-11 battery in a third session), minimizing the effect of some tasks on others.

Similarly, the finding is only applicable to the age group ranging from 6 to 8 years old, as the relation between cognitive and motor factors can change as result of age. Moreover, other factors not contemplated in this research could add explicative power to the model to explain mathematical performance, such as executive functions, math anxiety or socioeconomic status (Hentges, Galla & Wang, 2018; Kolkman et al., 2013; Maloney & Beilock, 2012). In fact, spatial skills, such as MR, are correlated with other cognitive skills that have not been tested in this study (such as working memory, Mammarella et al., 2013b), so, further research that contemplates abilities tightly associated with spatial abilities are necessary to isolate the specific contribution of spatial skills to mathematical performance.

Furthermore, the present study may have important educational and clinical implications, as knowing the key factors that underlie mathematical performance when students start their first steps in compulsory instruction, can be important to promote these factors at these first stages or to prevent possible low performances related with learning disabilities through a training that comprises the improvement of spatial and motor abilities. Although more research is necessary in order to establish the direct impact of training abilities in promoting performance in mathematical ability, the first step is to find what factors have a role in this age range (6–8 years old) specifically. Our findings help to constrain the spatial and motor abilities that can be underpinning mathematical success in primary school children and to differentiate which factors predict mathematical achievement according to age, by showing a stable and specific contribution, such as MR ability.

## Conclusion

The results of the present study highlight that visuospatial reasoning has a predictive power of mathematical performance at the beginning of school education, reducing its power as the child grows up. However, after controlling this factor, MR and fine motor skills specifically explained part of the success in mathematical ability in terms of written calculation and number ordering among 6 to 8 year-old children. Therefore, MR and motors skills arise as relevant factors in mathematical tasks in early years of school children.

##  Supplemental Information

10.7717/peerj.10095/supp-1 Supplemental Information 1 Scoring summary of block design test

10.7717/peerj.10095/supp-2 Supplemental Information 2 Raw data of the measures All measures used for statistical analysis to compare mathematical performance taking into account spatial and motor abilities in different developmental ages.

## Additional Information and Declarations

### Data Availability

The following information was supplied regarding data availability: The raw measurements are available as a Supplementary File.
