# Supplementary material for: Mathematical achievement: the role of spatial and motor skills in 6–8 year-old children
Source: PeerJ. 2020 Oct 6;8:e10095. doi: 10.7717/peerj.10095 (PMC7546220; doi:10.7717/peerj.10095)
Supplement: Supplemental Information 1 [file peerj-08-10095-s001.docx]

| Item | Time limit | Scoring |
| --- | --- | --- |
| 1 | 30 | Trial 1 = 2 points; Trial 2 = 1 point |
| 2 | 45 | Trial 1 = 2 points; Trial 2 = 1 point |
| 3 | 45 | Trial 1 = 2 points; Trial 2 = 1 point |
| 4 | 45 | 4 points |
| 5 | 45 | 4 points |
| 6 | 75 | 4 points |
| 7 | 75 | 4 points |
| 8 | 75 | 4 points |
| 9 | 75 | 1-10 sec = 7 points; 11-20 sec = 6; 21-30 sec = 5 points; 31-75 sec = 4 points |
| 10 | 75 | 1-10 sec = 7 points; 11-20 sec = 6; 21-30 sec = 5 points; 31-75 sec = 4 points |
| 11 | 120 | 1-30 sec = 7 points; 31-50 sec = 6; 51-70 sec = 5 points; 71-120 sec = 4 points |
| 12 | 120 | 1-30 sec = 7 points; 31-50 sec = 6; 51-70 sec = 5 points; 71-120 sec = 4 points |
| 13 | 120 | 1-30 sec = 7 points; 31-50 sec = 6; 51-70 sec = 5 points; 71-120 sec = 4 points |
| 14 | 120 | 1-30 sec = 7 points; 31-50 sec = 6; 51-70 sec = 5 points; 71-120 sec = 4 points |
